# Angiosarcoma of the Breast: Overview of Current Data and Multimodal Imaging Findings

**DOI:** 10.3390/jimaging9050094

**Published:** 2023-04-30

**Authors:** Marco Conti, Francesca Morciano, Claudia Rossati, Elisabetta Gori, Paolo Belli, Francesca Fornasa, Giovanna Romanucci, Rossella Rella

**Affiliations:** 1 UOC di Radiologia Toracica e Cardiovascolare, Dipartimento di Diagnostica per Immagini, Radioterapia Oncologica ed Ematologia, Fondazione Policlinico Universitario Agostino Gemelli IRCCS, Largo A. Gemelli 8, 00168 Rome, Italy; marco.conti@policlinicogemelli.it (M.C.);; 2 Facoltà di Medicina e Chirurgia, Università Cattolica Sacro Cuore, Largo F. Vito 1, 00168 Rome, Italy; francesca.morciano01@icatt.it (F.M.);; 3 UOSD Breast Unit ULSS9, Ospedale di Marzana, Piazzale Lambranzi, 1, 37142 Verona, Italy; claudiarossati@gmail.com (C.R.); francesca.fornasa@aulss9.veneto.it (F.F.);; 4 UOC Diagnostica per Immagini, Ospedale G.B. Grassi, Via Gian Carlo Passeroni, 28, 00122 Rome, Italy

**Keywords:** angiosarcoma, breast imaging, primary breast angiosarcoma, secondary breast angiosarcoma, mammography, ultrasound, magnetic resonance imaging, computed tomography, treatment, prognosis

## Abstract

Angiosarcoma of the breast is a rare breast cancer, which can arise de novo (primary breast angiosarcoma, PBA) or as a secondary malignancy (secondary breast angiosarcoma, SBA) as a result of a biological insult. In the latter case, it is usually diagnosed in patients with a previous history of radiation therapy following a conserving treatment for breast cancer. Over the years, the advances in early diagnosis and treatment of breast cancer, with increasing use of breast-conserving surgery and radiation therapy (instead of radical mastectomy), brought about an increased incidence of the secondary type. PBA and SBA have different clinical presentations and often represent a diagnostic challenge due to the nonspecific imaging findings. The purpose of this paper is to review and describe the radiological features of breast angiosarcoma, both in conventional and advanced imaging to guide radiologists in the diagnosis and management of this rare tumor.

## 1. Introduction

Angiosarcoma of the breast (BA) is a rare malignant breast cancer, representing around 0.04% of all breast cancers and 8% of breast sarcomas [1,2]. It can arise de novo (primary breast angiosarcoma, PBA) or develop after a biological insult, e.g., radiation therapy (secondary breast angiosarcoma, SBA) [3]. These two entities are clinically and pathologically different: PBA usually originates from breast parenchyma and may affect the skin, with different degrees of cutaneous and subcutaneous involvement; conversely, SBA originates from the skin previously included in an irradiation field or at its limits, in the so-called “twilight zone” where radiation is inhomogeneous, with a subsequent potential invasion of the underlying breast tissue [4,5,6,7,8]. They also differ for the median age of presentation: around 40 years for PBA and 70 years for SBA, with a median of 72 months postradiotherapy [5,9].

When breast-conserving treatment (BCT) was not so common, SBA was generally seen after mastectomy and axillary dissection in association with lymphedema, in the so-called Stewart–Treves syndrome [10,11,12]. Nowadays, it is mostly linked to a previous history of radiation therapy [10]. For this reason, in recent years, the number of diagnosed SBAs is increasing in parallel with the number of patients treated with breast-conserving surgery and adjuvant radiotherapy [3]. In the USA, the absolute risk of radiation-associated SBA is estimated at 7 per 100,000 person-years post-BCT [13].

The purpose of this paper is to describe the radiological features of this rare tumor and provide a comprehensive review of the available literature about its presentation in both conventional and advanced imaging to guide radiologists in its diagnosis and management.

## 2. Clinical Presentation

PBA and SBA are two different histopathological entities with different clinical presentations.

PBA is a rare entity, with unknown risk factors, which affects younger women. Most PBA presents as a palpable mass with rapid growth, with a mean tumor size ranging from 5.7 cm to 7.3 cm according to previously published studies [9,14,15], located within the mammary gland and which can extend to the skin causing ulceration [14]. Rarely, PBA presents with diffuse swelling of the entire breast, sensation of fullness or alteration of the breast shape [9,14]. PBA can also be associated with Kasabach–Merritt syndrome (KMS), a disease with a poor prognosis, more frequently diagnosed in children with giant hemangiomas [9,16,17]. KMS is characterized by thrombocytopenia, due to platelets sequestration in a large vascular lesion, and consumptive coagulopathy with a bleeding diathesis [18].

**Figure 1:**
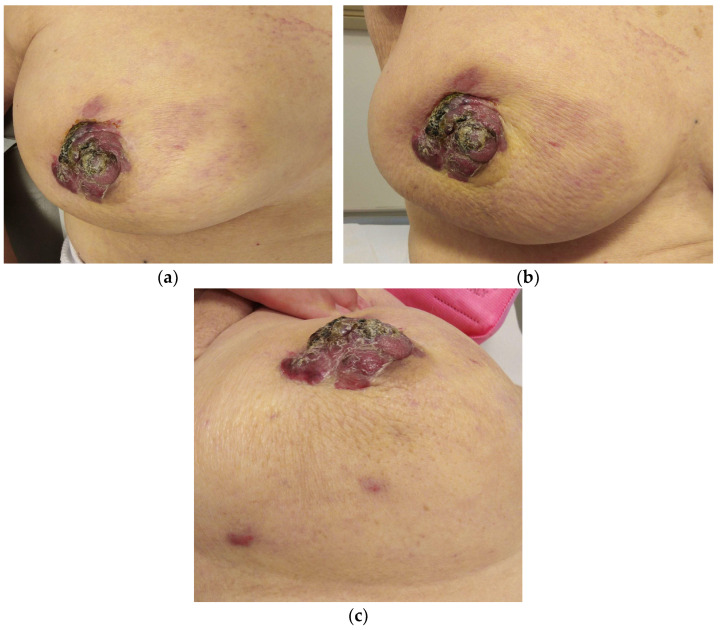
An 85-year-old patient with a history of carcinoma of the right breast treated with quadrantectomy and radiotherapy. (**a**–**c**) Clinical examination revealed papillary erythematous lesion, skin thickening, and multiple reddish nodules.

About SBA, it has cutaneous and/or subcutaneous localization [19,20,21,22] so it often presents with cutaneous changes and lesions, either in the chest wall or residual breast parenchyma, rather than a palpable mass. Cutaneous lesions are predominantly represented by erythematous or violaceous plaques, nodules, ecchymosis [6], skin discoloration, erythematous areas, bluish to reddish or black nodules, and edema [23] (Figure 1). Ulceration may or may not be present [19].

Concerning the dimension of skin lesions in SBA, Ginter et al. remarked that their extension is usually greater than 2 cm, helping to differentiate SBA from benign vascular tumors, which commonly are infracentimetric [19].

SBA diagnosis is often delayed because it usually arises in a postirradiated or postsurgical breast. Therefore, it could be difficult to differentiate it from post-actinic skin changes (i.e., radiodermatitis) [24]. Any cutaneous lesion in postirradiated residual breast parenchyma or chest wall should be further investigated in the suspicion of SBA.

For these reasons, clinical examination is crucial in the suspicion of BA and photographic documentation can be a helpful tool in this field.

## 3. Pathogenesis

The pathogenesis of angiosarcomas and especially BA is certainly a multifactorial process. BReast CAncer 1 and 2 (BRCA1 and BRCA2) are two of the most studied genes in the development of breast and ovarian cancer. These genes are involved in cellular homeostasis and protection against radiation-induced DNA damage, thus their connection with the development of SBA has been speculated; West et al. and Kadouri et al. reported some cases of SBA in BRCA1 and BRCA2 mutation carriers [25,26]. The transcription factor p53 and its main inhibitor MDM2 were linked to development of angiosarcomas both on an animal model [27,28] and in patients with BA, also associated with upregulation of vascular endothelial growing factor (VEGF) expression [29,30,31]. Amplification of MYC gene, which codes for a transcription factor, has been reported to be present in cases of secondary angiosarcoma [32,33]; Guo et al. hypothesized that Fms-related tyrosine kinase (FLT4), encoding a tyrosine kinase receptor for vascular endothelial growth factors, may be a potential candidate for this gene amplification [34]. Finally, the PIK3CA/AKT/mTOR pathway may also be directly or indirectly linked to the development of angiosarcoma and may represent a possible target for therapy [35].

PBA can also arise during pregnancy [36]. Nevertheless, the only publication that specifically investigated and reported the presence of steroid receptors (i.e., estrogens, progesterone, glucocorticoids) in these tumors evaluated only two cases of BA: the first case showed the expression of estrogens and progesterone receptors, the latter only the estrogens ones [37]. Conversely, the research of these hormonal receptors (estrogen and progesterone) in the published cases of PBA diagnosed during pregnancy resulted negative [38,39]. Further studies should be performed, with a larger population, to determine if hormonal receptors and thus endocrine aspects could be involved in PMA development during pregnancy.

## 4. Imaging Findings

### 4.1. Mammography

BA is a rare entity so only a few studies evaluated mammographic appearance of both primary and secondary BA.

**Figure 2:**
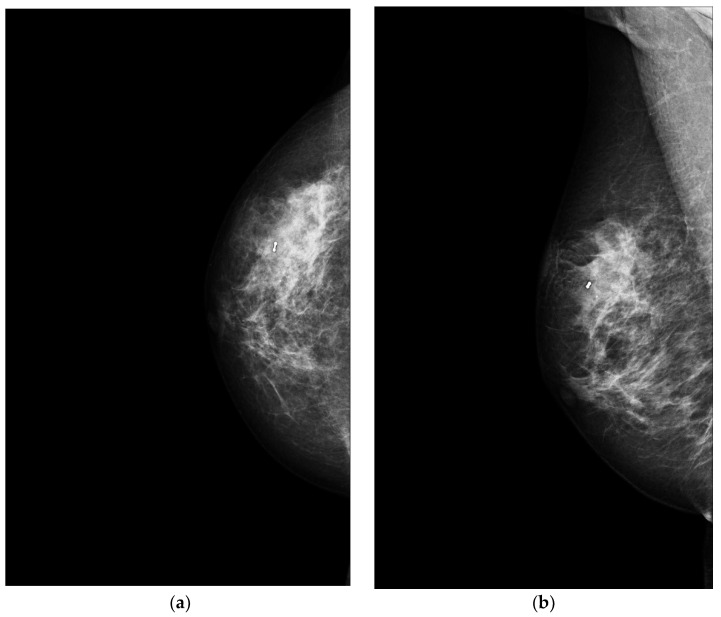
A 60-year-old patient with a history of gastric lymphoma treated 30 years previously. Clinical examination revealed an erythematous area on the right breast. (**a**) Craniocaudal and (**b**) mediolateral oblique mammograms of the right breast show an irregular mass. A core needle biopsy was performed with the simultaneous placement of a clip marker (white dot) and the presence of a primary BA was histologically proven.

Focusing on PBAs, Wu et al. investigated their features on mammography, concluding that they are nonspecific [40]. Indeed, the authors describe PBAs as lobulated or oval masses, large diffuse asymmetries with irregular density, alterations of breast trabecular structure, thickened local vessels, irregular densities of subcutaneous fat, and skin thickening. Nevertheless, no enlarged axillary lymph nodes were reported. The authors did not find associated microcalcifications: this could be explained by the origin of PBA from parenchymal rather than ductal structures (where the calcium is deposited) and/or by its rapid growth [40]. Another retrospective study published by Wang et al. investigated PBAs on 36 patients and agreed on the nonspecificity of their mammographic characteristics: circumscribed noncalcified masses were described in most of the patients and no remarkable findings were found in 43.8% of patients [14]. However, patients diagnosed with PBA were more likely to present with a clinically and radiographically detectable parenchymal mass than those with SBA (*p* < 0.002) [3]. Additionally, BA might manifest as a focal asymmetry and this can be explained by its infiltrating nature [1] (Figure 2).

**Figure 3:**
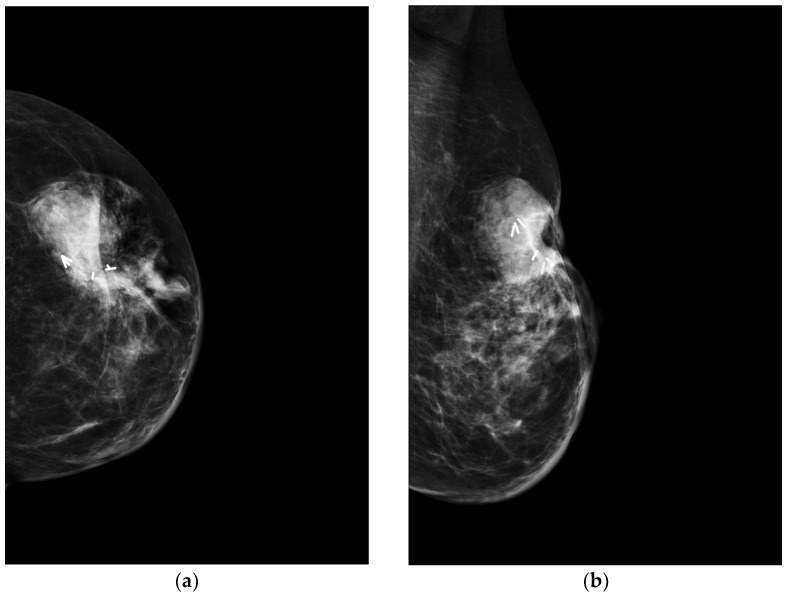
A 70-year-old patient with a history of carcinoma of the left breast treated with quadrantectomy and radiotherapy. Three years after completion of radiotherapy, clinical examination revealed a reddish cutaneous area and a palpable mass in the surgical scar location. (**a**) Craniocaudal and (**b**) mediolateral oblique mammograms of the left breast show a high density, irregular mass below the scar associated to skin thickening. The white lines represent the surgical clips released at the time of the quadrantectomy.

About SBA, its mammographic appearance can overlap postirradiation modifications of breast post-BCT, which include skin thickening, retraction, and architectural distortion of the breast parenchyma, or an ill-defined mass [5,41,42] (Figure 3).

**Figure 4:**
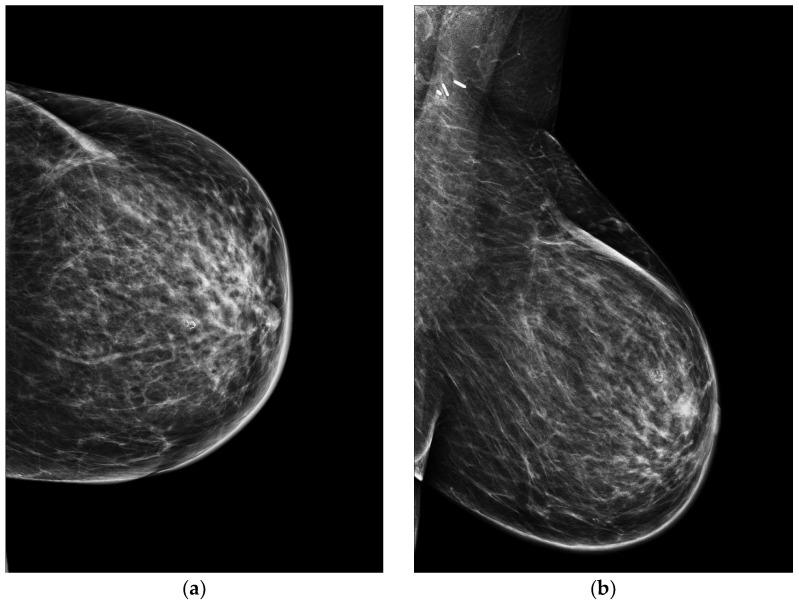
A 58-year-old patient with a history of carcinoma of the left breast treated with quadrantectomy and radiotherapy. Seven years after completion of radiotherapy, clinical examination revealed an erythematous lesion in the scar location. (**a**) Craniocaudal and (**b**) mediolateral oblique mammograms of the left breast show only skin thickening without mass or calcifications association. Due to the persistence of the cutaneous lesion, a punch biopsy was performed revealing an SBA.

**Figure 5:**
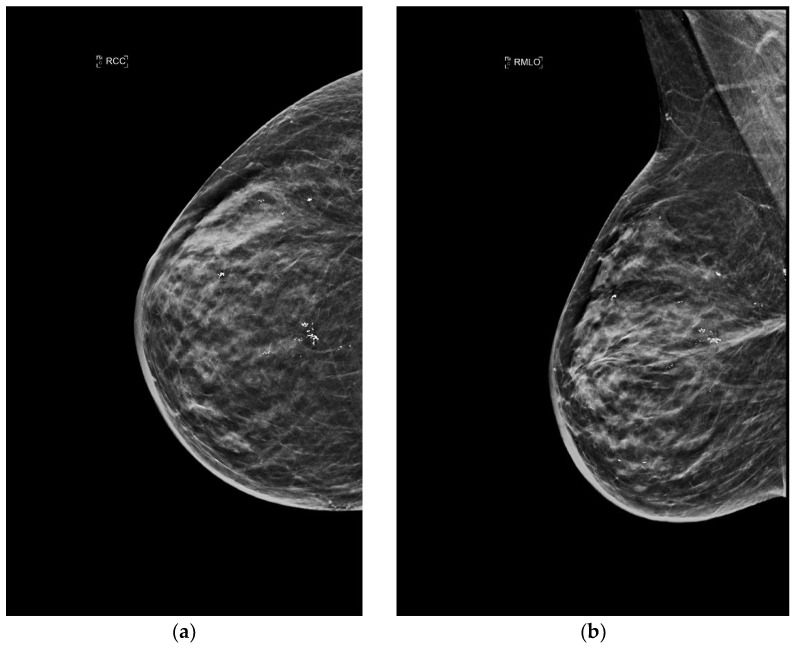
A 75-year-old patient with a history of lobular invasive carcinoma of the right breast treated with quadrantectomy and radiotherapy. Clinical examination revealed skin discolouring and teleangectasis five years after completion of radiotherapy. (**a**) Right craniocaudal (RCC) and (**b**) mediolateral oblique (MLO) mammograms show scar remodeling and skin thickening. No mass or suspicious calcifications were detected. A histological diagnosis of SBA was made with a punch biopsy.

However, it should be noted that mammography can give false negative results even after skin changes [43,44,45,46]. Indeed, some authors estimate that approximately 33% of radiation-associated SBA could present with a completely normal mammography [42]. Therefore, it is important to highlight that in the follow-up of women post-BCT, skin thickening is expected to decrease two years after radiotherapy [47]. Any increase in cutaneous thickness after this period should raise the suspicion of malignancy, for example, of carcinomatous mastitis or SBA. When this sign is detected on mammography, radiologists should reconsider the patient’s cancer history and carry out a clinical examination that can reveal the presence of skin discoloration, edema, skin papules, nodules, and/or vesicles [20,42] (Figure 4 and Figure 5).

A recently published multicentric study investigated the role of imaging in primary and secondary BA in a large geographical area and over a long interval of time (25 years). In this paper, authors found that SBA is more likely to manifest as skin changes rather than parenchymal findings (i.e., masses) [3].

To the best of our knowledge, there is only one published case that examined the characteristics of SBA on contrast-enhanced mammography. In this case, the patient had a history of BCT, and, at clinical examination, skin discoloration and multiple dermal lesions were found in the previously treated breast, with a mean size of about 8 cm. When compared with previous mammograms, the last one showed both skin and trabecular thickening, while recombined views highlighted a considerable subcutaneous nonmass enhancement. A targeted ultrasound (US) examination was performed which confirmed skin thickening with increased vascularity. A US-guided core needle biopsy was performed and the presence of SBA was histologically proved [48].

### 4.2. Ultrasound

US examination is an essential tool in breast imaging, which allows the characterization of lesions, with high sensitivity also in dense breasts, as in younger women, in whom the sensitivity of mammography is lower and the presence of breast lesions, such as BA, may be obscured [49].

**Figure 6:**
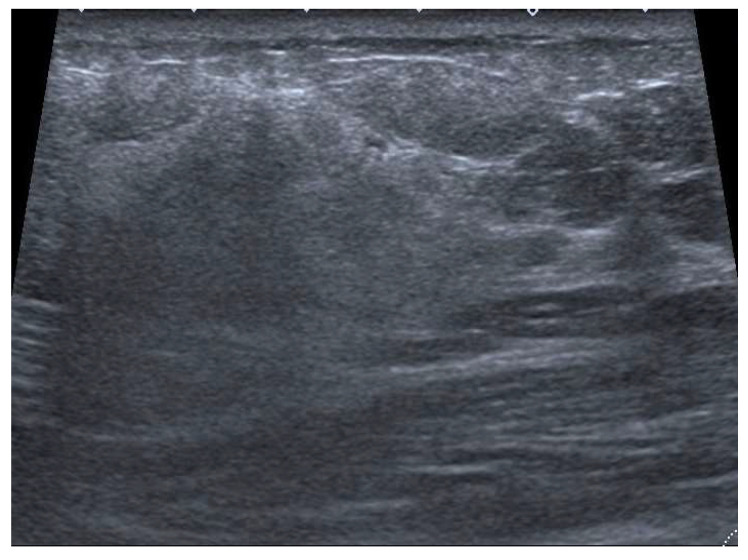
A 60-year-old patient with a palpable mass. US image of a PBA characterized by a voluminous heterogeneous hyperechoic mass, with non-circumscribed margins, with associated skin thickening.

**Figure 7:**
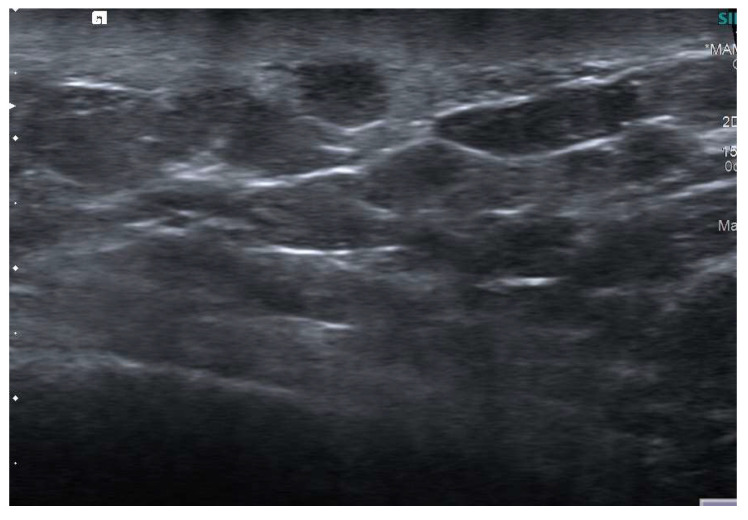
A 58-year-old patient with a history of carcinoma of the left breast treated with quadrantectomy and radiotherapy. US image of an SBA characterized by a diffuse skin thickening and an oval-shaped hypoechoic lesion with circumscribed margins.

**Figure 8:**
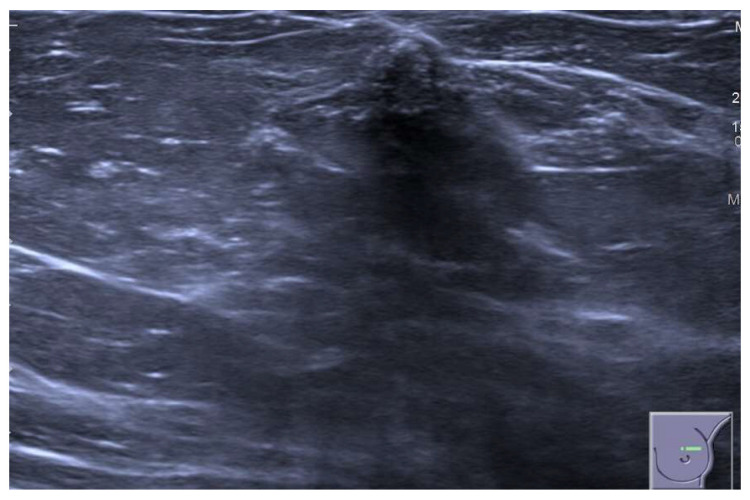
A 75-year-old patient with a history of lobular invasive carcinoma of the right breast treated with quadrantectomy and radiotherapy. US image of an SBA characterized by skin thickening and an irregular mass with indistinct margins in the retroareolar area. A US-guided core needle biopsy was performed, revealing a single focus of atypical cellular proliferation. Then, a punch biopsy was performed and an SBA was histologically proven.

A study published by Yang et al. retrospectively reviewed the US features of 21 BAs: most of them appeared as solid masses with an oval shape and circumscribed margins, without posterior acoustic phenomena, and, in a minority of cases, with lobular shape and indistinct or microlobulated margins. In addition, almost half of mass lesions are hypoechogenic, while the others may show hyperechogenicity or mixed features: this could be explained by the vascular nature of the tumor with the presence of multiple interfaces of vascular channels [1] (Figure 6, Figure 7 and Figure 8).

Focusing on the differential diagnosis with other hyperechoic breast lesions, it should be highlighted that the majority of them are benign. These include: lipoma, angiolipoma, haematoma, seroma, fat necrosis, silicone granuloma, sebaceous or epidermal inclusion cyst, abscess, pseudoangiomatous stromal hyperplasia (PASH), galactocele or lactating adenoma, ductal ectasia, and apocrine metaplasia [50]. On the other hand, even other malignant lesions can be hyperechoic: infiltrating ductal or invasive lobular carcinomas, but also some uncommon tumors, such as lymphoma or metastatic lesions [50].

Another US feature may be the presence of anechoic components, linked to hemorrhage and necrosis [51].

When Color doppler imaging is performed, BA presents an increased vascularity [1,52] or variable internal blood flow [3].

However, as previously described, BA can also be characterized by cutaneous changes and lesions: in these cases, US often shows nonspecific findings or demonstrates skin edema or thickening with associated suspicious hypoechoic intracutaneous lesions, as reported by Salminen et al. [53].

To date, only two case reports investigated the features of BAs on contrast-enhanced ultrasound (CEUS), both about PBA: the first one, described by Yang et al., was characterized by peripheral irregular and rapid enhancement with slow washout, and with a central area of nonenhancement, probability due to perfusion defects [54]; the second one was described by Qin et al. as a mass with scattered and inhomogeneous early enhancement, suggesting an increased vascularization within the mass when compared with normal breast parenchyma [55].

### 4.3. Magnetic Resonance Imaging

Given the possibility of false negatives and nonspecific findings on conventional imaging, magnetic resonance imaging (MRI) could be useful in the differential diagnosis and in the evaluation of the extent of the disease when BA is suspected [1,53,56,57]. Moreover, MRI was proven to be the most sensitive technique for the detection of SBA associated with radiation therapy [53].

**Figure 9:**
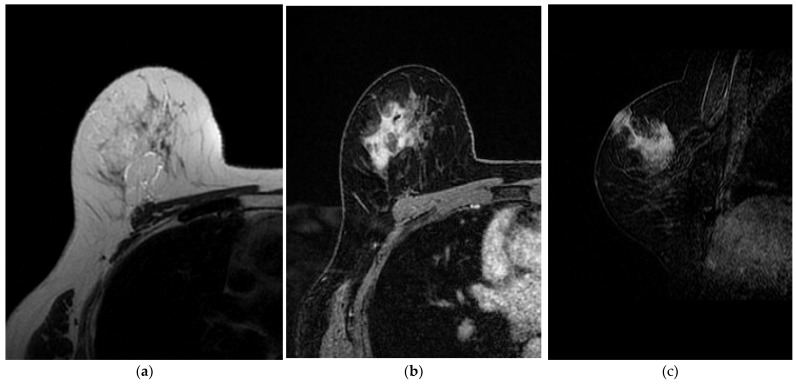
A 60-year-old patient with PBA. (**a**) T2-weighted, (**b**) axial 3D gradient echo T1-weighted post-contrast and (**c**) sagittal 3D gradient echo T1-weighted post-contrast images show an irregular mass, with non-circumscribed margins, characterized by heterogeneous enhancement, with skin thickening and invasion.

In a study conducted on thirteen women, BA showed both heterogeneous and slightly or significantly high homogeneous with hypointense necrotic or cystic components on T1-weighted images and inhomogeneous high signal on T2-weighted (w) images. About half of them presented as hemorrhagic lesions characterized by hyperintensity on both T1-w and T2-w images or with a hemosiderin ring (resulting from long-term hemorrhage). On post-contrast images, a vivid heterogeneous enhancement was demonstrated [40]. In particular, the kinetic enhancement is strictly linked to tumor grade: the kinetic curve shows a rapid enhancement with rapid washout in the highest grades, while a plateau or a persistent enhancement is seen in the lower ones [58,59,60,61,62,63,64]. In addition, high-grade tumors may also present hemorrhage or vascular lakes, displayed as focal areas of hyperintensity on T1-w images [58] (Figure 9).

Focusing on radiation-associated SBA, the published papers reported various MRI morphological appearance: a retrospective study analyzed the MRI features of 16 cases finding out that the majority of them (81.3%) showed diffuse heterogeneous skin enhancement, sometimes associated with T2-hypointense nodular skin foci of rapid early arterial enhancement with washout [56]. Authors hypothesized that postradiation inflammatory changes may explain skin thickening with diffuse high signal on T2-w images whilst tumoral cells would actually manifest as hypointense foci [56].

**Figure 10:**
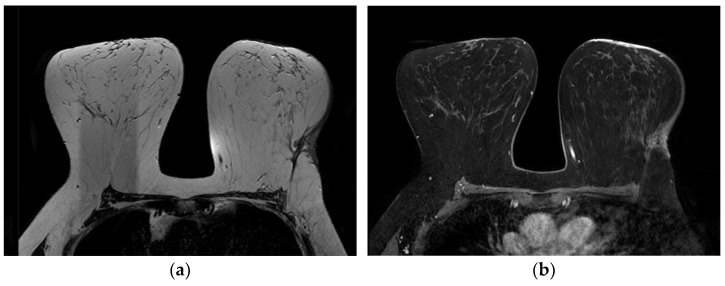
A 58-year-old patient with a history of carcinoma of the left breast treated with quadrantectomy and radiotherapy. (**a**) T2-weighted and (**b**) 3D gradient echo T1-weighted post-contrast images show skin thickening near the scar and late contrast enhancement. Punch biopsy revealed SBA.

**Figure 11:**
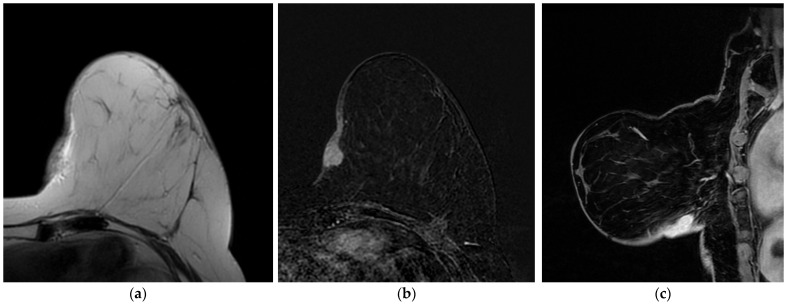
A 48-year-old patient with a history of left breast cancer treated with quadrantectomy and radiotherapy. (**a**) T2-weighted, (**b**) axial 3D gradient echo T1-weighted post-contrast and (**c**) sagittal 3D gradient echo T1-weighted post-contrast images show skin thickening and an oval-shaped mass, with circumscribed margins, characterized by homogenous enhancement. US-guided core needle biopsy reveals SBA.

However, other MRI appearance of SBA were described: in a case report by Sanders et al., a infracentimetric enhancing skin lesion was described [57], while Vuille-dit-bille et al. reported a nonspecific thickening and enhancement of the skin, without focal nodularity or masses in the underlying breast parenchyma [65]; in three MRI examinations performed at diagnosis, Bentley et al. found a parenchymal mass associated to nonmass enhancement and cutaneous thickening or nodularity/masses and nipple enlargement [3]. Finally, Bentley et al. also analyzed the MRI appearance of recurrent disease in two patients previously treated for BA, reporting a skin nodule in the first case and a chest wall and subcutaneous nodules in the second one [3] (Figure 10 and Figure 11).

### 4.4. Computed Tomography

Computed tomography (CT) has no broad application in the diagnosis of breast lesions but, in this setting, it is essential for staging purposes.

**Figure 12:**
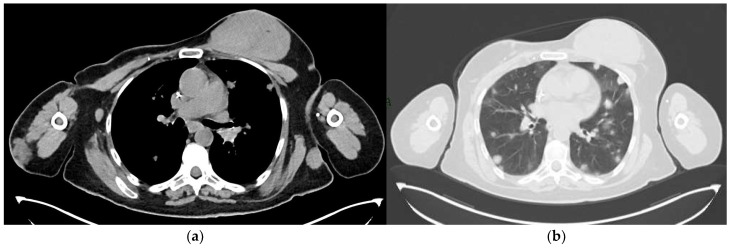
A 65-year-old patient with a history of PBA treated with right mastectomy, right axillary lymph node dissection and radiotherapy of the right axilla. A 2-year follow-up with CT scan revealed metastatic disease: (**a**) Mediastinal window showing a voluminous mass in the left breast, an enlarged lymph node in the right axilla, and a nodular thickening in the subcutaneous soft tissues of the right arm and of the left lateral chest wall. (**b**) Parenchymal window showing numerous bilateral pulmonary nodules.

CT can be useful to find distant localizations of disease in case of metastatic spread. In particular, lung parenchyma is the most frequent localization, but metastasis were reported also in liver, skin, bone, central nervous system, spleen, lymph nodes, ovary, and heart [3,61,66,67,68] (Figure 12).

**Figure 13:**
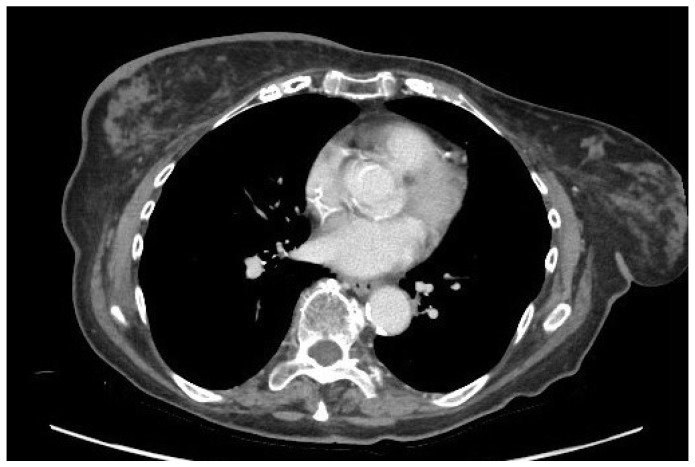
A 75-year-old patient with a history of lobular invasive carcinoma of the right breast treated with quadrantectomy and radiotherapy. Mediastinal window of a follow-up CT scan shows skin thickening of the right breast.

In addition, Salminen et al. retrospectively reviewed the available imaging of a nationwide cohort of patients with SBA, finding out that CT has similar sensitivity to MRI in the evaluation of SBA, warning that both techniques could be falsely negative [53] (Figure 13).

Moreover, taking into account that BA might present with compromised local conditions (i.e., breast swelling, skin induration, or ulcerations), it could be uncomfortable for patients to undergo MRI examination, due to prone position and lengthy acquisition time; in this setting, CT could be used as a valid alternative.

### 4.5. PET-CT

**Figure 14:**
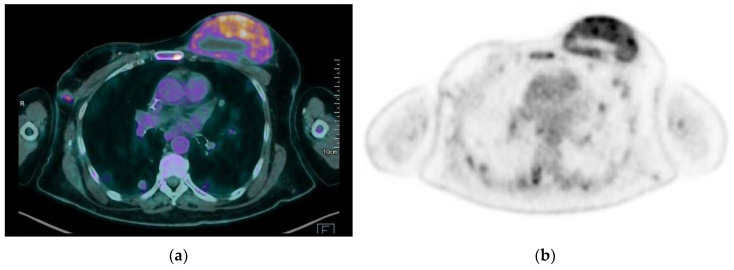
A 65-year-old patient with a history of PBA treated with right mastectomy, right axillary lymph node dissection, and radiotherapy of the right axilla. A 2-year follow-up with ^18^F FDG PET-CT scan revealed metastatic disease: (**a**,**b**) hypermetabolic lesions are observed in breasts, lungs, and bones.

There is a lack of knowledge about the role of positron emission tomography-computed tomography (PET-CT) in breast angiosarcoma. The few case reports available in the current literature show that PBA and SBA are avid of F-18 fluoro-2-deoxy-D-glucose (FDG) in both primary and metastatic localizations [4,69,70] (Figure 14).

A report of thirteen lesions found that the two subtypes are visually and statistically different in maximum standardized uptake value (SUV_max_); nevertheless, lesions with the highest SUV_max_ presented a worse prognosis regardless of the subtype [70].

To our knowledge, no study investigated the role of PET/MRI.

Although further research is needed, this imaging technique may be helpful for the initial staging of BA and to evaluate therapeutic response in follow-up by demonstrating the resolution of metabolic activity.

## 5. Histopathology

Immunohistochemistry is very important in this setting to differentiate BA from invasive carcinomas. The endothelial markers of angiosarcoma are CD 34, CD 31, and factor VIII. Higher Ki67 index is linked with poor prognosis [71].

After surgical resection, histopathological and immunohistochemical analysis of the entire specimen are mandatory and represent the gold standard for diagnosis: indeed, fine needle aspiration and core needle biopsy may result falsely negative for malignancy (with an estimated 37% of cases), because of the existence of well differentiated histotypes as well as the presence of necrotic and fatty tissue or hemorrhage [40].

Differential diagnoses concerning low-grade BA include angiolipoma, hemangioma, and benign proliferative lesions; instead for the higher grades, mastitis, fibromatosis, but especially to invasive mammary carcinoma [66].

BAs can present with different grades: low-grade angiosarcoma, consisting of well-formed anastomosing vascular channels, intermediate grade angiosarcoma (characterized by an important neoplastic vascular growth), and high-grade angiosarcoma, that show localized necrosis, infarction, and hemorrhage [36,40].

## 6. Treatment and Prognosis

Given the rarity of this cancer, there is no real consensus on treatment and the management varies depending on the institution, ranging from simple wide excision to radical mastectomy [2,21,72,73,74,75]. Like all soft tissue sarcomas, BA presents better outcomes when managed in a multidisciplinary team [76].

The majority of the authors agree that nodal involvement is not common in BA [21,72,75,77,78,79,80]. Nevertheless, while lymphatic dissemination is unusual, early metastases can usually be found due to preferential hematogenous pathways, potentially involving any anatomic location, especially lungs [81,82].

Treatments vary according to the stage of the disease (localized vs. metastatic) [76]. About localized disease, the gold standard is surgery with R0 (microscopically margin-negative) resection: as BA are infiltrative tumors, wide margins are recommended; positive margins are linked to worse prognosis with a high risk of local recurrence [76]. Indeed, R0 resections are proven to have significantly higher survival rates in radiation-associated SBA when compared to R1 (microscopically irradical) or R2 (macroscopically irradical) resections [83]. Regarding radiation-associated BA, some authors considered surgical margins to be safe when reaching 2–4 cm [84,85,86]; however, a case report reported a successful local control with just 1 cm of clear margins [46].

The effectiveness of adjuvant chemotherapy is still not clear as published results are discordant [87,88] and represented by case reports, which mainly suggest its efficacy for locally recurrent and metastatic SBA [89,90,91], or small-scale studies, which found an improved survival in patients with SBA but not in PBA [92].

Although some authors suggested that chemotherapy regimens, like gemcitabine, doxorubicin, and taxanes, have cytotoxic effects in BA, their efficacy on long-term survival remains uncertain [93,94].

Focusing on SBA, many studies found that survival and local recurrence rates were poor even when surgery was combined with standard adjuvant chemotherapy [83,95,96,97] A retrospective study conducted by Lagrange et all. reported no significant difference in survival rates when comparing patients treated with surgery alone with those treated with surgery and chemotherapy [98]. Nevertheless, several studies found that adjuvant chemotherapy can be beneficial after surgery with wide clear margins [23,72,99,100,101,102,103]. Rosen et al. reported that adjuvant chemotherapy improved disease-free survival rate of higher-grade BA [72].

The use of radiotherapy is also controversial, in particular in radiation-associated SBA: in these cases, skin was already irradiated, raising concerns about an increased toxicity [89]. However, Palta et al. evaluated the outcomes of 14 cases of radiation-associated BA treated with hyperfractionated and accelerated radiotherapy, with or without surgery, finding encouraging results in terms of local control, disease-free likelihood, and overall survival [104].

About BA prognosis, it is influenced by margin involvement after surgical treatment, histological grade, and tumor dimension. Gutkin et al. reviewed 58 patients with nonmetastatic BA finding that 5-year overall survival was 73.7% for PBA and 63.5% for SBA, with an improved local control when resection margins were superior to 5 mm [92]. Regarding radiation-associated BA, the 5-year survival rate and disease-free survival rate were reported around 27–48% [105,106] and 35%, respectively [107]. An important prognostic factor was the number of cutaneous lesions: the 2-year survival rate increases from 0% to 50% depending on the presence of multiple or single lesions [20,108]. Moreover, low-grade tumors are linked to longer disease-free survival intervals [98,109]. Brady et al. described lesion dimension (with a cutoff of 5 cm), metastatic spread, and surgery as the most important prognostic factors, with impact on mortality rate [95].

## 7. Conclusions

Breast angiosarcoma is a rare and highly malignant breast tumor. Unfortunately, only a few studies were published on this topic, mainly represented by case reports and small-scale studies. For these reasons, BA often represents a diagnostic challenge for breast radiologists. It is important to be familiar with clinical presentation and imaging features of BA to achieve early diagnosis. When BA is suspected, anamnestic information should be properly collected, with particular regard to previous breast cancer history.

## Data Availability

No new data were created.
